# Body dissatisfaction, drive for thinness, and psychopathological symptoms in preadolescents who use Instagram

**DOI:** 10.1007/s40519-025-01766-9

**Published:** 2025-07-17

**Authors:** Silvia Cimino, Carlos A. Almenara, Luca Cerniglia

**Affiliations:** 1 Department of Dynamic, Clinical and Health Psychology, Sapienza, University of Rome, Rome, Italy; 2 School of Health Sciences, Universidad Peruana de Ciencias Aplicadas, Lima, Peru; 3 Faculty of Psychology, International Telematic University Uninettuno, Rome, Italy

**Keywords:** Instagram, Preadolescents, Drive for thinness, Body dissatisfaction, Psychopathological symptoms, Social media

## Abstract

**Background:**

Preadolescence is a critical developmental stage marked by the start of significant physical and cognitive changes posing youths at risk for psychopathology. This study explores the association of Instagram usage with body dissatisfaction, drive for thinness and psychopathological symptoms among preadolescents.

**Methods:**

We recruited 232 female preadolescents aged 9–10 years and their mothers using a snowball sampling technique. Participants were divided into two groups based on their Instagram addiction levels: no addiction (Group 1) and addiction (Group 2). Measures included the Eating Disorders Inventory-Referral Form (EDI-3-RF), Instagram Addiction Scale (IAS-15), and Child Behavior Checklist (CBCL).

**Results:**

Results showed that Group 2 had significantly higher scores in body dissatisfaction, drive for thinness, emotional reactivity, and withdrawal compared to Group 1.

**Conclusions:**

Limitations include the small, homogenous sample, reliance on self-report measures, and cross-sectional design, which limits causal inference. Future research should include more diverse samples, longitudinal designs, and a broader assessment of social media use to better understand these complex relationships. Addressing these limitations will enhance our understanding and contribute to developing effective interventions to support the mental health of preadolescents in the digital age.

*Level III*: Evidence obtained from well-designed cohort or case–control analytic studies.

## Introduction

It is well recognized that adolescents focus on their bodies [1]. Especially, the physical maturation and changes taking place during that developmental stage are associated with the start of puberty and the accompanying hormonal shifts, which are key indicators of the transition from childhood to adolescence. Adolescence is associated with significant brain growth, especially in the prefrontal cortex, which controls executive processes including decision-making, impulse control, and risk appraisal. Though cognitive skills dramatically improve throughout this time, self-control and long-term thinking-related brain areas are still developing. The inability of teenagers to control their emotions and conduct may be caused by this developmental gap [2].

Concerns with body image and changes in psychological well-being can result from biological changes such as changes in body fat and the emergence of secondary sexual traits [3]. Teenagers may be predisposed to different risks for the beginning of psychopathology due to the interaction between biological changes and psychosocial variables [4]. Many young people frequently must deal with the emergence of dysfunctional eating-related symptoms or eating disorders [5–9]. To the best of our knowledge, very few studies have focused on very young adolescents, with most of the worldwide literature on this topic primarily studying subjects starting from their early adolescence (11–13 years). Although menarche in females and puberty in males have been shown to occur earlier in recent years, some scientists have recommended that the concept of adolescence itself should be updated [10]. The same authors acknowledge that it could be debatable to classify a 9–10 year old child as an adolescent. However, other research suggests that children as young as 9–10 years old may be regarded as pre- or early adolescents since they have traits associated with later adolescence (impulsivity, experience seeking, propensity to rely on peers rather than family as a reference group, etc.) [11]. These traits in pre-early teens frequently reflect fragility and instability in their identities rather than a cognitive or emotional development. Some experts have connected premature and widespread social media use with the emotional/behavioral functioning of early teenage children [12, 13].

On the other hand, the use of digital technologies has grown among very young people in recent years, and they are very active users of social media [14]. For example, studies such as EU Kids Online 2020 [15], have revealed that 9–11 years old Italians spend an average of 93 min online and 23% of them visit social networking sites daily or more often. Instagram's minimum age requirement is 13 years old, and thus children under age 13 are typically underrepresented in official statistics. Nevertheless, Instagram is the favorite social media platform of young users as reported by 25.5% of girls aged 16–24, and the fourth most used social media platform with 2 billion monthly active users worldwide [16]. In Italy, Instagram has around 27 million users and most of them (52.9%) are female [17].

It has been proposed that adolescents' development is now digitally mediated since digital media have been described as super-peers [18], and are significant in the formation of early adolescent ideas and attitudes. Youths typically consume large quantities of videos portraying peers while doing various activities such as singing, dancing, sporting, and so on. In addition, to illustrate their talents in different realms, these videos are much related to the use of body and its appearance [19, 20]. As Instagram is a visually-oriented platform, it is centered around sharing images and curated content, playing a pivotal role in shaping preadolescents' perceptions of their own bodies. A study conducted by Anixiadis et al. [21] demonstrated that exposure to idealized images on Instagram led to higher levels of body dissatisfaction among adolescent girls. The visual nature of Instagram, combined with the portrayal of unrealistic beauty standards, has the potential to adversely affect the self-esteem and body image of preadolescents.

In conclusion, it appears that pre-early adolescents, who were traditionally classified as being in the latency developmental stage, are increasingly displaying adolescent-like behaviors without demonstrating a corresponding emotional maturation. This phenomenon has been linked to the use of social media, such as Instagram, in recent years. However, at the same time, the body's centrality—once a feature of adolescence and now present in pre-early adolescents as well—seems to bring types of psychopathology to the fore, although Anorexia Nervosa or subthreshold disordered eating symptoms in subjects younger than twelve years of age are relatively understudied [22, 23]. To the best of our knowledge, however, very few studies have focused on such young populations addressing disordered eating behaviors, psychopathological symptoms, and the use of Instagram.

Consistently with these considerations and to fill the gap in previous literature, this study aimed to evaluate the levels of psychopathological symptoms, and disordered eating symptoms in a group of 9–10 years old youths who addictively used Instagram, comparing them with a group of peers who were not addicted to the social network.

## Methods

### Participants and procedure

We used a consecutive snowball sampling technique to gather a sample from the general population through social media platforms like Facebook and announcements on online psychology research websites. The inclusion criteria were: (1) no reported psychiatric diagnoses in the participants; and (2) not undergoing any medical or psychological treatment. We recruited 242 female adolescents aged 9–10 years and their mothers. The decision to recruit only female adolescents for this study stems from their heightened vulnerability to body dissatisfaction and the drive for thinness, as research consistently shows that these issues are more prevalent among girls [24, 25]. Female adolescents disproportionately use Instagram, an image-based platform, and are thus more exposed to unrealistic beauty ideals that can exacerbate psychopathological symptoms like anxiety and depression [26]. Gender-specific research allows for deeper exploration of these dynamics without the confounding effects of gender differences, thereby increasing methodological clarity [27]. Furthermore, adolescent girls are more likely to internalize social comparison behaviors, making them a critical demographic for this analysis [28]. Addressing this population directly contributes to targeted interventions aimed at mitigating mental health risks in a digital age [29]. By focusing exclusively on female adolescents, the study provides valuable insights into a vulnerable group, advancing both academic knowledge and practical applications in mental health and media literacy. We excluded from the sample: adolescents whose parents did not consent to let their offspring participate (n = 8); and preadolescents with a psychiatric diagnosis (n = 2). As a result, the final sample included 232 female preadolescents and their mothers. Most participants were Caucasian (97.3%) and 82% came from families with an average annual household income between EUR 28,000 and 55,000 (average socio-economic status). Additionally, 88% of the participants were from intact family units. The authors confirm that all procedures complied with the ethical standards of the relevant national and institutional committees on human experimentation and the Helsinki Declaration of 1975, as revised in 2008. All procedures involving human subjects were approved by the Ethical Committee of La Sapienza, University of Rome, prior to the study (protocol code 0000809 of 09/10/2020). All parents or guardians of the participants and minors provided written informed consent. Anonymity and privacy of personal information were assured. Mothers and preadolescents completed a series of report-form (mothers) and report-form measures (preadolescents) and a specially designed questionnaire to collect sociodemographic information.

### Measures

Body satisfaction and disordered eating were assessed through the Eating Disorders Inventory-*Referral Form* (EDI-3-RF; [30, 31] for the Italian version). The EDI-3-RF is a 25-item brief self-report measure, derived from the EDI-3 (which contains 91 items) and designed to measure eating disorder risk. It can be administered in non-clinical or clinical settings and, as indicated by the scale’s author and by other studies (e.g., [32]), the measure can be administered to subjects as young as eleven years old. The EDI-3-RF uses a 6-point Likert scale (1 = always, 6 = never) forming the Drive for Thinness (e.g., “I think about dieting”), Bulimia (e.g., “I eat moderately in front of others and stuff myself when they’re gone”), and Body Dissatisfaction subscales (e.g., “I think that my stomach is too big”). Internal consistency of EDI-3-RF in this study was 0.80.

### Use of Instagram

Instagram Addiction Scale (IAS-15): The 15-item IAS [33] was used to assess the risk of Instagram addiction. Items (e.g., “How often do you try to cut down the amount of time you spend on Instagram and fail?”) are rated on a six-point Likert scale from 1 (never) to 6 (always) with scores ranging from 15 to 90. The higher the score, the greater the risk of Instagram addiction. The cut-off points are defined as: no addiction (15–37), mild addiction (58–38), moderate addiction (73–59), and severe addiction (more than 73). The scale comprises two sub-factors: social effect (eight items: e.g., “How often do you prefer the excitement of Instagram instead of being with your close friends?”) and compulsion (seven items: e.g., “How often do you try to cut down the amount of time you spend on Instagram and fail?”). The social effect subfactor refers to negative consequences of addictive Instagram use in relation to individuals’ real-life social relationships. The compulsion subfactor refers to the increasing need for Instagram use, the frequency of forgetting about time while on Instagram, and the avoidance of real-life troubles using Instagram [34]. For the purposes of this study only the total score of the scale was used, using the above cut-offs to define two groups: no addiction to Instagram (Group 1) and addiction to Instagram (Group 2).

### Psychopathological symptoms

The Child Behavior Check-List—CBCL 6–18 is a dimensional (as opposed to categorical) measure of emotional and behavioral problems. It contains several scales that are arranged hierarchically, a structure that emerged through factor analysis (see [35]). Each level of the hierarchy reflects the breadth of coverage of a variety of emotional and behavioral problems. At the top of this hierarchy are the broadly focused *Internalizing Domain* and the *Externalizing Domain*. The *Internalizing Domain* is a broad measure of emotional problems: it is an aggregate of anxiety and depression symptoms that subsumes three more narrowly focused *syndrome* scales: *Anxious/Depressed, Withdrawn/Depressed,* and *Somatic Complaints*. The *Externalizing Domain* is an aggregate measure of behavioral problems and includes the *Rule Breaking Behavior* and *Aggressive Behavior* syndrome scales. A *Total Problems* score is also available, which quantifies the overall extent of both emotional and behavioral problems based on responses to all CBCL items including those on the three remaining syndrome scales: *Social Problems, Thought Problems*, and *Attention Problems*. The parent or primary caregiver completes the CBCL. Each item is scored on a 3-point Likert scale (0 = *Not True,* 1 = *Somewhat or Sometimes True,* or 2 = *Very True or Often True*) to describe the child's behavior during the preceding 6 months. In this study internal consistency was 0.75–0.92.

## Data analyses

All analyses were performed using IBM SPSS software, version 26. Preliminary statistical analyses were conducted using descriptive statistics (reliability of the measures, frequencies, mean scores) and to define Group 1 (no Instagram addiction) and Group 2 (Instagram addiction). ANOVAs for repeated assessments were used to compare the outcomes on all variables for the Group 1 and Group 2. *p* values less than < 0.001 were considered significant. Cohen’s [36] criteria and a power of 0.85 were followed to interpret effect size.

## Results

Table 1 shows mean and standard deviation values of Group 1 and Group 2 on the IAS scale. The effect size, indicated by eta-squared (η^2^ = 0.71), suggests a large proportion of the variance in IAS scores is explained by group membership.

**Table 1:** Means (standard deviation) of the child’s IAS

	Group 1	Group 2	η^2^
	M (SD)	M (SD)	
IAS	23.32 (3.11)	42.55 (3.45)	0.71**

**<0.001

Regarding the CBCL subscales, there were notable differences between the groups. As for the Emotional Reactivity subscale Group 2 had significantly higher scores (*M* = 5.28, *SD* = 1.95) compared to Group 1 (*M* = 2.84, *SD* = 1.55), with a large effect size (η^2^ = 0.63, *p* < 0.001), indicating greater emotional reactivity in Group 2. Group 1 scored higher (*M* = 5.96, *SD* = 1.88) than Group 2 (*M* = 4.91, *SD* = 1.89) at Anxious/Depressed subscale, with a non-significant effect size (η^2^ = 0.23). On the Somatic Complaints subscale both groups had similar scores, with Group 1 (*M* = 2.55, *SD* = 2.51) and Group 2 (*M* = 2.47, *SD* = 1.38), resulting in a non-significant effect size (η^2^ = 0.19). On the Withdrawn subscale Group 2 had significantly higher scores (*M* = 6.09, *SD* = 1.36) compared to Group 1 (*M* = 2.65, *SD* = 1.86), with a large effect size (η^2^ = 0.64, *p* < 0.001). Conversely, on Phobic Anxiety and Aggressive Behavior the two groups showed small to moderate differences, with Group 2 generally scoring (not significantly) higher in Phobic Anxiety and slightly higher in Aggressive Behavior. As for the Internalizing Problems subscale Group 2 had higher scores (*M* = 19.86, *SD* = 2.69) compared to Group 1 (*M* = 17.68, *SD* = 2.57), with a large effect size (η^2^ = 0.77, *p* < 0.001), whereas on the Externalizing Problems subscale Group 1 (*M* = 12.20, *SD* = 2.57) and Group 2 (*M* = 11.36, *SD* = 2.27) had similar scores, with a non-significant effect size (η^2^ = 0.18).

For the EDI-3SF subscales, significant differences were observed. For the Drive for Thinness (D-T) subscale, Group 2 scored significantly higher (*M* = 5.28, *SD* = 1.95) than Group 1 (*M* = 2.84, *SD* = 1.55), with a large effect size (η^2^ = 0.81, *p* < 0.001). In terms of the Bulimia (B-U) subscale, both groups had similar scores, with Group 1 (*M* = 5.16, *SD* = 1.88) and Group 2 (*M* = 4.91, *SD* = 1.89), yielding a non-significant effect size (η^2^ = 0.23). Regarding the Body Dissatisfaction (B-D) subscale, with Group 1 showed significantly higher scores (*M* = 4.55, *SD* = 1.51) than Group 2 (*M* = 2.47, *SD* = 1.38), resulting in a moderate/large effect size (η^2^ = 0.61) (Tables 2, 3).

**Table 2:** Means (standard deviation) of the child’s CBCL subscales

	Group 1	Group 2	η^2^
	M (SD)	M (SD)	
E-R	2.84 (1.55)	5.28 (1.95)	0.63**
A-D	5.96 (1.88)	4.91 (1.89)	0.23
S-C	2.55 (2.51)	2.47 (1.38)	0.19
WIT	2.65 (1.86)	6.09 (1.36)	0.64**
A-P	2.54 (1.41)	3.14 (1.61)	0.29
A-B	2.69 (1.77)	2.82 (2.38)	0.28
INT	17.68 (2.57)	19.86 (2.69)	0.77**
EXT	12.20 (2.57)	11.36 (2.27)	0.18

η^2^: eta-squared, E-R: emotional reactivity, A-D: Anxious/Depressed, S-C: somatic complaints, WIT: withdrawn, A-P: phobic anxiety, A-B: aggressive behavior, INT: internalizing problems, EXT: externalizing problems

**p < .001

**Table 3:** Means (standard deviation) of the child’s EDI-3SF subscales

	Group 1	Group 2	η^2^
	M (SD)	M (SD)	
D-T	2.84 (1.55)	5.28 (1.95)	0.81**
B-U	5.16 (1.88)	4.91 (1.89)	0.23
B-D	2.55 (2.51)	2.47 (1.38)	0.19

η^2^: eta-squared, D-T: drive for thinness, B-U: bulimia, B-D: body dissatisfaction

**p < .001

## Discussion

The widespread adoption of social media platforms has brought about profound changes in various aspects of society, including its impact on individual well-being. Particularly concerning is the influence of social media, specifically Instagram, on the psychological health of preadolescents. This study aimed to investigate the levels of body dissatisfaction, drive for thinness and psychopathological symptoms among 9–10 year-old youths who excessively use Instagram, compared to their peers who do not. Our findings revealed that excessive Instagram usage is significantly associated with higher body dissatisfaction and an increased risk of drive for thinness among preadolescents. The findings align with Möri et al. [37], who reported a clear association between social media use and disordered eating behaviors and cognitions in young adolescents. It also aligns with the results of Meier and Gray [38], who found that elevated appearance exposure on Facebook was correlated with weight dissatisfaction, drive for thinness, and self-objectification among adolescent girls, indicating that the negative impacts of social media on body image are not confined to a single platform but may be a broader phenomenon associated with visually-oriented social networks. However, conflicting literature exists. Mustikasari and Rahayu [39] found no significant relationship between Instagram usage and body image among adolescents, suggesting that the impact of social media might vary based on individual differences or contextual factors. McLean et al. [40] indicated that while sharing self-images on social media was linked to higher body dissatisfaction and eating concerns, general media exposure did not show the same effect. These discrepancies could be attributed to differences in study populations, methodologies, and the specific aspects of social media use examined.

Our results also showed significant differences in emotional and behavioral problems between addicted Instagram users (Group 2) and non-addicted users (Group 1). Group 2 had significantly higher scores on the Emotional Reactivity and Withdrawn subscales of the Child Behavior Check-List (CBCL), suggesting greater emotional and social difficulties. Specifically, Group 2 exhibited greater emotional reactivity compared to Group 1, and also scored higher on the Withdrawn subscale than Group 1. These findings indicate that Instagram addicted users are more prone to emotional instability and social withdrawal. Conversely, on the Anxious/Depressed subscale, Group 1 scored higher than Group 2, although this difference was not statistically significant. This suggests that non-addicted users may experience higher levels of anxiety and depression, which could be attributed to factors unrelated to Instagram use.

In this regard, Verrastro et al. [41] highlighted that adolescents who frequently edit and upload photos on Instagram internalize beauty standards more, leading to increased anxiety and discomfort with their body image, which can manifest in higher emotional reactivity. The phenomenon of upward social comparison, where individuals assess themselves against those perceived as superior, might also be at the basis of this phenomenon [42]. Peers and influencers both contribute to the upward social comparison process among preadolescents. Influencers, often celebrated for their curated and aspirational lifestyles, serve as models of comparison that can amplify feelings of inadequacy and dissatisfaction among young users [25]. For example, Nadeem et al. [43] established that exposure to social media influencers was associated with greater body dissatisfaction and appearance-related comparisons. In sum, social media comparisons that lead to negative self-evaluations can exacerbate emotional difficulties which could explain why Instagram-addicted users (Group 2) exhibited higher emotional instability.

On the other hand, studies have found that depressed adolescents commonly display social disengagement and withdrawal and turn to social media for engaging in social comparison and feedback-seeking [44]. However, in this study addicted Instagram users (Group 2) scored lower in anxiety/depression (although the differences were not significant) and higher in social withdrawal. Therefore, this finding can be hardly attributed to anxiety or depression levels in this sample. A more plausible explanation can be the *displacement hypothesis* that suggests that offline interactions are replaced by the online ones [45], as well as the fear of missing out (FoMO, i.e., the concern that others are having rewarding experiences without you; [46]), which has been related to greater social media use and poorer well-being (see [47]). Further research is warranted to unveil this relationship among preadolescent girls.

## Strengths and limits

This study has some limitations that should be considered when interpreting the results. First, the sample size was relatively small and consisted exclusively of female preadolescents from a specific socio-economic background, primarily Caucasian, which may limit the generalizability of the findings. The homogeneity of the sample restricts our ability to apply the results to a more diverse population, including males and individuals from various racial, ethnic, and socio-economic backgrounds. Moreover, the cross-sectional design of the study limits the ability to draw causal inferences about the relationship between Instagram use and body dissatisfaction. While associations can be identified, it is not possible to determine the direction of these relationships or to confirm whether Instagram use directly leads to increased body dissatisfaction and disordered eating behaviors, or if individuals with pre-existing body dissatisfaction are more likely to engage with Instagram. In addition, the study relied on self-report measures for data collection, which can be subject to biases such as social desirability and recall bias. Participants may underreport or overreport their Instagram usage and symptoms of body dissatisfaction and disordered eating due to these biases. Additionally, the accuracy of self-reported data on social media use can be questionable, as participants might not accurately remember or may intentionally misreport their online activities.

Further, the study focused specifically on Instagram usage without considering the potential influences of other social media platforms such as Tik Tok. Given that adolescents often use multiple social media platforms, the findings might not fully capture the broader social media landscape and its associations with body image and psychological well-being. Future research should consider a more comprehensive assessment of social media usage across various platforms. Furthermore, there may be other unmeasured variables that contribute to body dissatisfaction and disordered eating behaviors, such as family dynamics, peer influences, or pre-existing psychological conditions. These confounding variables were not controlled for in the study, which may affect the validity of the findings. Moreover, the recruitment of mothers in this study is both a strength and a limitation. As a strength, mothers provided critical insights into their children’s emotional and behavioral functioning through the CBCL, adding depth and objectivity to the findings [35]. Their participation also ensured ethical oversight and a broader contextual understanding of family dynamics. However, reliance on maternal reports could introduce bias, as their perceptions may not fully capture the children’s experiences or be influenced by their own attitudes and behaviors [13]. Furthermore, excluding paternal or other caregiver perspectives may limit the comprehensiveness of the family context examined in this study [48]. Finally, social media platforms and usage patterns are continually evolving. The specific features and functions of Instagram, as well as user engagement with these features, may change over time. As such, the findings of this study may not be applicable to future iterations of the platform or to future generations of users. Despite these limitations, the study provides valuable insights into the association between Instagram use, drive for thinness, body dissatisfaction and psychopathological symptoms among preadolescents. Future research should address these limitations by including larger and more diverse samples, utilizing longitudinal designs to establish causality, and considering the influence of multiple social media platforms. Additionally, incorporating objective measures of social media use and controlling for potential confounding variables would enhance the robustness of the findings. By acknowledging and addressing these limitations, researchers can better understand the complex relationship between social media use and psychological well-being, ultimately contributing to the development of effective interventions to support the mental health of preadolescents in the digital age.

## What is already known on this subject?

Adolescence is a critical period marked by significant physical, cognitive, and emotional changes that contribute to heightened body image concerns and vulnerability to psychopathology. Research has shown that social media platforms, particularly Instagram, play a central role in shaping adolescents' self-perception, as they are highly visual and promote idealized beauty standards. Studies have demonstrated that exposure to idealized images on Instagram is associated with increased body dissatisfaction, drive for thinness, and disordered eating behaviors, particularly among adolescent girls. The role of social media as a "super-peer" suggests that it may influence young users' attitudes and self-evaluation similarly to real-life peer interactions. While most studies have focused on adolescents aged 11 and older, limited research has examined these effects in younger populations, such as preadolescents aged 9–10 years. Given the increasing digitalization of childhood and the early adoption of social media, there is a growing need to investigate how Instagram addiction may impact body image and psychological well-being in preadolescent girls. 

## What does this study add?

This study extends previous research by examining the relationship between Instagram addiction and body image concerns in preadolescent girls (9-10 years old), a population that has been largely understudied in this context. Unlike prior studies that primarily focused on adolescents, this paper highlights how younger children are already engaging with social media and experiencing its psychological effects. The findings suggest that Instagram addiction is significantly associated with increased body dissatisfaction and internalization of beauty ideals, even at this early developmental stage. Additionally, the study explores the mediating role of psychological distress, demonstrating that the link between Instagram use and body image concerns is partially driven by underlying emotional vulnerabilities. By focusing on preadolescents, this research underscores the need for early interventions and parental guidance to mitigate the negative impact of social media on youths’ mental health. 

## Data Availability

Raw data are available at reasonable request to the authors.
